# Threshold size criterion to suspect malignant supraclavicular lymph node < 10 mm in esophageal cancer

**DOI:** 10.1186/s13244-025-01929-3

**Published:** 2025-03-05

**Authors:** Yue Huang, Jingsai Du, Qian Li, Tiantian Fan, Zhaoqi Wang, Funing Chu, Jing Li, Bing Li, Xiong Yang, Renzhi Zhang, Ihab R. Kamel, Yang Zhou, Zhen Li, Jinrong Qu

**Affiliations:** 1 Department of Radiology, The Affiliated Cancer Hospital of Zhengzhou University & Henan Cancer Hospital, Zhengzhou, China; 2 Department of Radiology, The First Affiliated Hospital of Henan Polytechnic University & The Second People’s Hospital of Jiaozuo, Jiaozuo, China; 3 Department of Radiology, Xinyang Central Hospital, Xinyang, China; 4 Department of Ultrasound, The Affiliated Cancer Hospital of Zhengzhou University & Henan Cancer Hospital, Zhengzhou, China; 5 Radiology Department, Harbin Medical University Cancer Hospital, Harbin, China; 6 Department of Radiation Oncology, The Affiliated Cancer Hospital of Zhengzhou University & Henan Cancer Hospital, Zhengzhou, China; 7 Department of Diagnostic Radiology, National Cancer Center/National Clinical Research Center for Cancer/Cancer Hospital, Chinese Academy of Medical Sciences and Peking Union Medical College, Beijing, China; 8 Department of Radiology, Anschutz Medical Campus, University of Colorado Denver, Aurora, CO USA; 9 Radiology Department, Tongji Hospital, Tongji Medical College, Huazhong University of Science and Technology, Wuhan, China

**Keywords:** Supraclavicular lymph node, Esophageal squamous cell carcinoma, Computed tomography, Short-axis diameter, Diagnostic accuracy

## Abstract

**Objectives:**

To determine the threshold size for predicting metastasis of supraclavicular lymph nodes (SCLNs) < 10 mm on axial and multiplanar reconstruction CT in esophageal squamous cell carcinoma (ESCC).

**Methods:**

This retrospective, multicenter study received approval from three institutional review boards, which waived informed consent. Patients with ESCC had ultrasound-guided fine-needle aspiration biopsy (US-FNAB) for SCLNs, with contrast-enhanced CT performed within 2 weeks prior to US-FNAB. A CT and ultrasound radiologist jointly analyzed images to identify and mark biopsied SCLNs < 10 mm on CT, followed by two blinded radiologists who independently measured short-axis diameter (SAD), long-axis diameter (LAD), short diameter of multiplanar reconstruction (SD-MPR), long diameter of multiplanar reconstruction (LD-MPR) and the intra-class correlation coefficient (ICC) was analyzed. Center 1 included 220 SCLNs as the training set, and Centers 2 + 3 included 75 SCLNs as the validation set. The optimal cutoff value was determined using receiver operating characteristic (ROC) curves.

**Results:**

In the training and validation sets, 31.8% (70/220) and 32.0% (24/75) of SCLNs were positive. ICC for SAD was excellent (ICC = 0.847). The area under the receiver operating characteristic curve of SAD was 0.832 in the training set, higher than others, with a cutoff value of > 6 mm, resulting in sensitivity, specificity, positive predictive value, negative predictive value, accuracy of 77.1%, 80.7%, 65.0%, 88.3%, 79.1%, respectively. In the validation set, these metrics were 87.5%, 74.5%, 61.8%, 92.7%, 81.0%, respectively.

**Conclusion:**

SAD on CT can suspect metastasis of SCLN < 10 mm in ESCC patients, with a threshold size of > 6 mm.

**Clinical relevance statement:**

Determining the threshold size criterion on CT images may enhance the prediction of supraclavicular lymph node metastasis in esophageal squamous cell carcinoma patients, thereby benefiting diagnostic and therapeutic strategies.

**Key Points:**

- Supraclavicular lymph nodes < 10 mm in esophageal carcinoma are indeterminate for malignancy.
- Supraclavicular lymph nodes > 6 mm are highly suspicious for malignancy.
- The metastasis status of supraclavicular lymph nodes is critical for staging esophageal carcinoma.

**Graphical Abstract:**

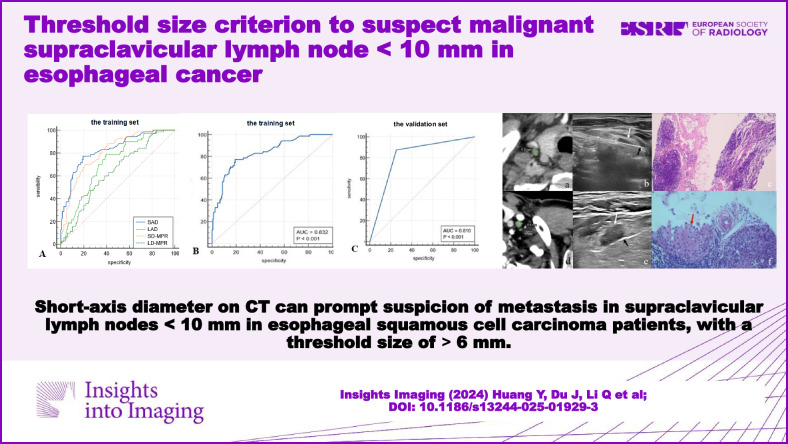

## Introduction

Esophageal cancer (EC) ranks eighth globally, predominantly as esophageal squamous cell carcinoma (ESCC) in Asia [1–3]. According to the American Joint Committee on Cancer (AJCC) 8th edition, supraclavicular lymph nodes (SCLNs) are classified as distant metastases (M1) [4]. Notably, the recent 12th edition of the Japanese classification of EC (JEC-12) has reclassified SCLNs as M1 [5, 6], differing from the previous 11th edition, which categorized them as regional LNs [7, 8]. This reclassification is pivotal, as SCLN involvement often precludes curative treatments [9]. Prior studies indicated that in EC patients, when at least one regional LN is positive and SCLNs are negative, the 5-year survival rate is 40.4%. However, with SCLN metastasis, this rate drops significantly to 24.1% [10].

Ultrasound-guided fine-needle aspiration biopsy (US-FNAB) is the favored diagnostic approach for evaluating SCLN metastasis in EC, despite its invasiveness, with reported sensitivity of 72% and an accuracy of 97% [11, 12]. Non-invasive imaging methods such as ultrasound (US), positron emission tomography (PET), and contrast-enhanced computed tomography (CT) are primarily used for LN metastasis assessment, each with limitations [4]. Specifically, US has a low specificity of 20% [13]. 18F-FDG PET/CT exhibits similar diagnostic performance with enhanced CT for evaluating EC metastatic LNs, with accuracies of 68% and 63% [4], respectively. Recent research has highlighted the superior efficacy of the novel tracer 18-FAPI over 18F-FDG in the diagnosis of metastatic LNs in 48 lung cancer patients, with an accuracy of 73% [14]. However, no studies have yet reported on the effectiveness of 18-FAPI in diagnosing LNs in EC.

Contrast-enhanced CT remains the primary non-invasive modality for assessing LN status in EC patients [4]. The short-axis diameter (SAD) measurement of LNs, a widely accepted standard in RECIST 1.1, defines involvement as SAD ≥ 10 mm [15]. However, SAD measurement of LNs cannot reflect the overall size and shape of LNs accurately [16]. Multiplanar reconstruction (MPR) can display the overall size and shape of LNs and is also easy to operate in clinical practice.

Previous studies have demonstrated unsatisfactory sensitivity when using this criterion to evaluate EC LN metastases [17], primarily due to the neglect of LNs with SAD < 10 mm (small LNs) [18–21]. Moreover, study has shown that only 8.0–37.5% of metastatic LNs in EC patients have a SAD ≥ 10 mm [19]. Wan et al highlighted that small, unresected LNs independently predict poor prognosis and high recurrence rates in ESCC patients, emphasizing the importance of assessing the metastatic status of small LNs [22]. These studies predominantly focus on regional and abdominal LNs [19, 23–25], overlooking SCLNs, and few studies have reported a definite threshold size criterion for small SCLNs.

Therefore, this study aims to determine the optimal threshold for predicting the metastasis status of SCLNs with SAD < 10 mm on contrast-enhanced axial and MPR CT images in ESCC patients.

## Materials and methods

### Participants

This retrospective study received approval from three institutional review boards, with waived patient written informed consent. ESCC patients who underwent US-FNAB of the SCLNs initially detected during the initial diagnosis, treatment or follow-up were included in this study. These patients underwent contrast-enhanced CT scanning within 2 weeks prior to US-FNAB. Inclusion criteria of US-FNAB included enlarged SCLNs with a short axis greater than 5 mm, rounded morphology, absence of an echogenic hilus, diffuse hypo-echogenicity, and/or intranodal necrosis [11, 26]. Data included consecutive patients from January 2021 to December 2023 in Center 1, from June 2018 to September 2022 in Center 2, and from January 2021 to December 2023 in Center 3. Inclusion criteria comprised (1) ESCC patients with the first discovery of SCLNs with SAD < 10 mm undergoing US-FNAB, (2) availability of contrast-enhanced CT images within 2 weeks before US-FNAB of SCLN, and (3) absence of other concurrent malignancies. Exclusion criteria included (1) no definitive histopathological information of the target SCLNs due to insufficient biopsy tissue, (2) incomplete CT images of the target SCLNs or unclear boundaries with adjacent tissues (other LNs and muscular tissue), and (3) presence of two or more LNs in the supraclavicular area, with uncertainty regarding the targeted LN identified on CT images after consultation with the sonographer.

**Fig. 1:**
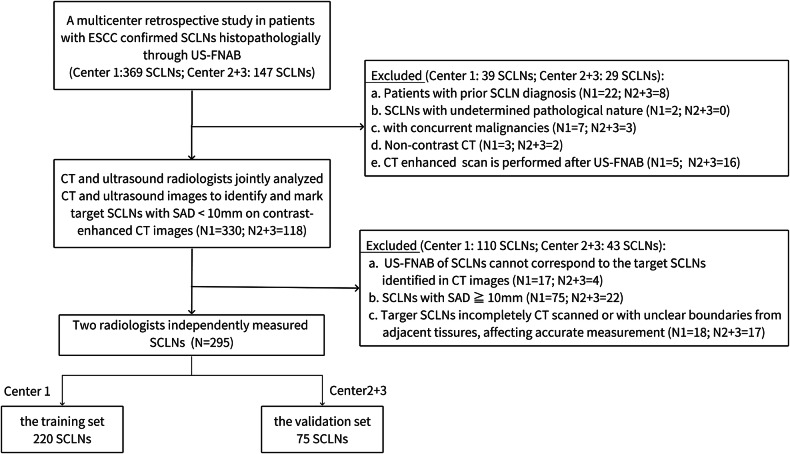
Flowchart of data collection

A total of 283 patients were included in this study. Center 1 enrolled 210 patients, 10 of whom had bilateral small SCLNs, resulting in 220 SCLNs as the training set. Centers 2 and 3 enrolled 73 patients, 2 of whom had bilateral small SCLNs, totaling 75 SCLNs as the validation set. The flowchart of patient enrollment is depicted in Fig. 1.

### CT protocol and parameters

All patients were positioned headfirst in the supine position and underwent contrast-enhanced CT scans from the supraclavicular area to the costophrenic angle, utilizing reconstruction section thicknesses of 0.625 mm, 1 mm, and 1.25 mm, in adherence to the 8th edition of AJCC/UICC staging for EC. Comprehensive conventional staging was conducted by analyzing chest, abdomen, pelvis CT, endoscopic ultrasound (EUS), and US of the neck, complemented by PET/CT. Detailed CT scanning parameters in the three centers are provided in Supplementary Tables S1, S2, and S3, respectively. The standard scanning protocols for the three centers are as follows:

Center 1: All patients received 1.5 mL/kg of body weight of iohexol injection (320 mg/mL, GE Healthcare Shanghai Co., Ltd.) at a rate of 2–3 mL/s via a high-pressure injector (Shenzhen Boon Medical Supply Co., Ltd.). Single-phase contrast-enhanced scans were performed during the venous phase, with a delay of 50–60 s.

Center 2: All patients received 1.5 mL/kg of body weight of iohexol injection (350 mg/mL, GE, Boston, USA) at a rate of 2–3 mL/s via a high-pressure injector. The arterial phase scan commenced 20–30 s after the contrast injection, the venous phase scan was performed at 60–70 s after injection, and the delayed phase scan was conducted at 180 s after injection. The images were exported in DICOM format, with the venous phase images used for further analysis.

Center 3: All patients received 1.5 mL/kg of body weight of iohexol injection (Optiray, 350 mg/mL; Mallinckrodt, Canada) at a rate of 3 mL/s via a high-pressure injector (Stellant; Medrad, USA). The bolus tracking technique was used to automatically trigger arterial phase, venous phase, and delayed phase acquisition at 20–25 s, 70–80 s, and 3–5 s after the threshold enhancement of the thoracic descending aorta reached 150 HU. The images were exported in DICOM format, with the venous phase images used for further analysis.

### Measurement

In accordance with JEC-12, the SCLNs, including the lower internal deep cervical LNs, are situated within the supraclavicular fossa and extend to the lower border of the cricoid cartilage. The medial boundary is defined by the medial border of the carotid sheath, with distinct demarcation between the left and right sides [5, 6].

**Fig. 2:**
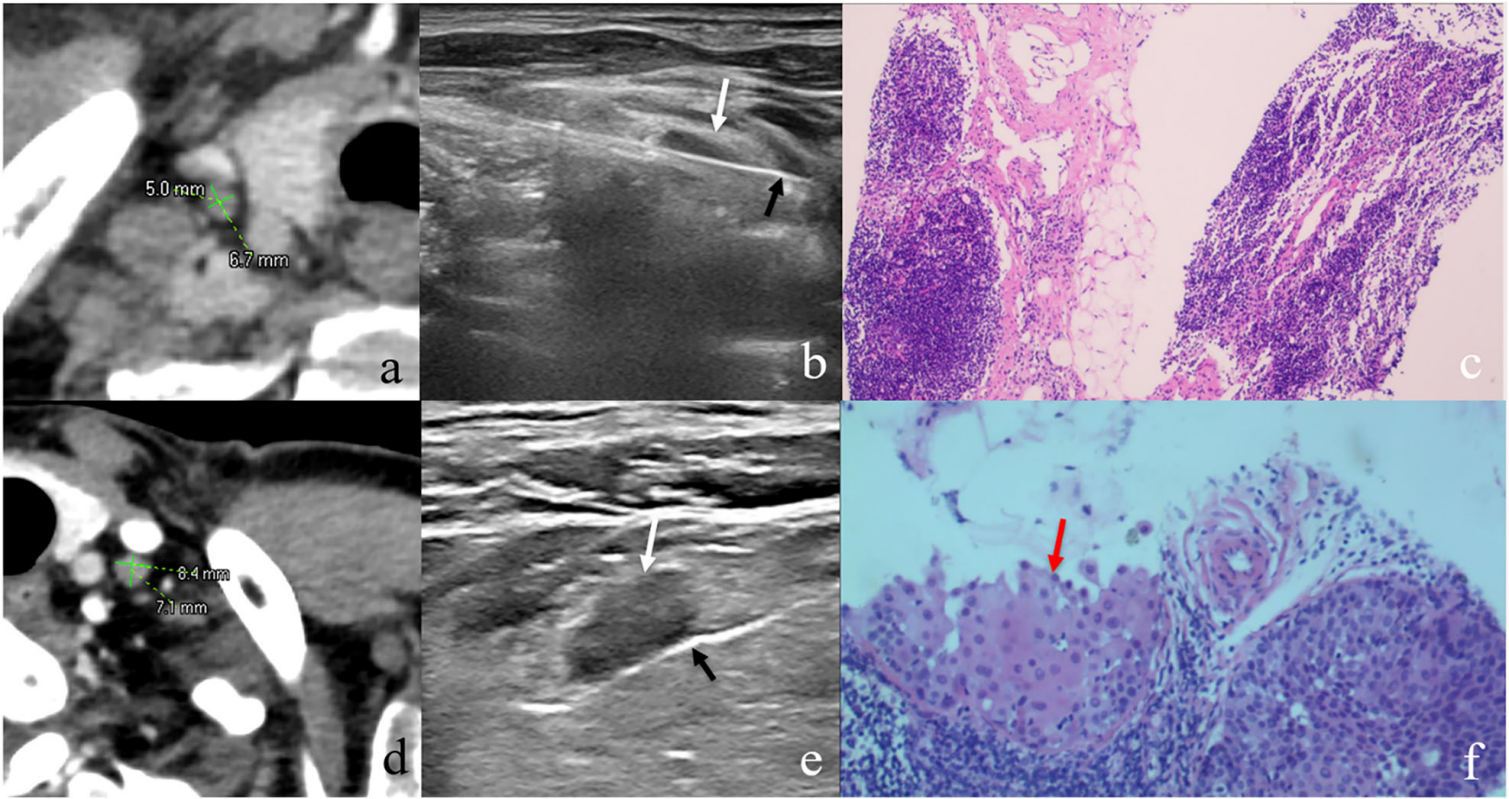
A 73-year-old male with ESCC. Upper row: **a** Enhanced CT image shows a small SCLN in the right with SAD of 5.0 mm; **b** US-FNAB (black arrow denotes the fine-needle) of the right SCLN (white arrow); **c** H&E-stained section at × 40 magnification confirms absence of cancer cells. A 49-year-old male with ESCC. Lower row: **d** Enhanced CT image shows a small SCLN in the left with SAD of 7.1 mm; **e** US-FNAB of the left SCLN; **f** H&E-stained section at × 100 magnification confirms cancer infiltration (red arrow) consistent with squamous cell carcinoma

CT and US images were jointly and retrospectively analyzed by a radiologist with 8 years of experience in CT interpretation and another ultrasound radiologist with 10 years of experience in US-FNAB. They identified the target SCLNs for US-FNAB with SAD < 10 mm and marked them on the CT images. Subsequently, two independent radiologists (with 8 and 10 years of experience in chest imaging) blinded to the histopathological information, independently measured the SAD, long-axis diameter (LAD), short diameter of multiplanar reconstruction (SD-MPR), long diameter of multiplanar reconstruction (LD-MPR) of the target SCLN derived from the training set (measurements were rounded to one decimal place) using the picture archiving and communication system. To ensure precise measurement of all the diameters of LNs on enhanced CT images, we employed a strategy of enlarging the images. This magnification process helps ensure accurate and consistent measurements, thereby minimizing measurement errors to the greatest extent possible (Fig. 2).

### Statistical analysis

Statistical analysis was conducted using SPSS 25.0 and MedCalc (version 20.022-64 bit) software. Descriptive statistics were presented as mean ± standard deviation (SD) or as counts and percentages. For continuous variables with normal distribution, *t*-tests were employed to assess differences between two groups, whereas Mann–Whitney U tests were used for continuous variables with non-normal distribution. For categorical or qualitative information, chi-square tests were utilized. A two-tailed *p*-value of 0.05 was considered statistically significant.

The intra-class correlation coefficient (ICC) was calculated to analyze inter-reader measurement consistency. ICC values ranging from 0 to 0.39 indicate poor correlation, 0.4 to 0.59 indicate moderate correlation, 0.6 to 0.75 indicate good correlation, and > 0.75 indicate excellent correlation [27].

ROC analysis was performed using MedCalc (version 20.022-64 bit) to determine the AUC for the training set, with the aim of establishing the optimal cutoff value for small SCLNs in EC. Sensitivity, specificity, positive predictive value (PPV), negative predictive value (NPV), and accuracy were derived accordingly. Subsequently, the optimal SAD was applied to the validation set to obtain the AUC, and sensitivity, specificity, PPV, NPV, and accuracy.

## Results

### Clinical characteristics

The detailed clinical characteristics of the patients are presented in Table 1. A total of 220 small SCLNs were collected in the training set, and 75 small SCLNs in the validation set. Gender distribution: Male (161/210, 76.6%) in the training set, (62/73, 84.9%) in the validation set. Ages ranged from 49 to 78 years old in the training set, and from 39 to 74 years old in the validation set. Clinical data analysis revealed no significant statistical differences between the training set and the validation set in terms of age, gender, primary tumor location, clinical T stage of the primary tumor, location of small SCLNs, status of small SCLNs and reconstruction section thickness.

**Table 1:** Patient characteristics

Characteristics	Training set *N* = 220	Validation set *N* = 75	*p*-value
Age (years)^a^	64.4 ± 8.3	62.5 ± 8.2	0.398
Gender^b^			0.158
M	171 (77.7%)	64 (85.3%)	
F	49 (22.3%)	11 (14.7%)	
Location of primary tumor^b^			0.219
Cervical esophagus	2 (0.9%)	0 (0%)	
Upper thoracic	41 (18.6%)	16 (21.3%)	
Middle thoracic	109 (49.5%)	27 (36.0%)	
Lower thoracic	67 (30.5%)	31 (41.3%)	
Esophagogastric junction	1 (0%)	1 (1.3%)	
cT staging of primary tumor^b^			0.835
T1	23 (10.5%)	6 (8.0%)	
T2	41 (18.6%)	14 (18.7%)	
T3	94 (42.7%)	36 (48.0%)	
T4	62 (28.2%)	19 (25.3%)	
Location of small SCLNs^b^			0.737
Left	131 (59.5%)	43 (57.3%)	
Right	89 (40.5%)	32 (42.7%)	
Status of small SCLNs^b^			0.977
Positive	70 (31.8%)	24 (32.0%)	
Negative	150 (68.2%)	51 (68.0%)	
SAD^a^			0.871
Positive SCLNs	7.7 ± 1.5 mm	7.9 ± 1.5 mm	
Negative SCLNs	5.8 ± 1.4 mm	5.3 ± 1.5 mm	
Metastatic SCLNs^c^			
SAD < 5 mm	6% (3/49)	5% (1/20)	
10 mm ≥ SAD ≥ 5 mm	39% (67/171)	42% (23/55)	
Reconstruction section thickness^b^			0.504
0.625 mm	9 (4.1%)	21 (4.1%)	
1 mm	120 (54.6%)	35 (60.3%)	
1.25 mm	91 (41.3%)	19 (35.6%)	

*p-va*lue was calculated with one-way analysis of variance *p*-value was calculated with χ^2^ test or Fisher’s exact test. cT staging is short for clinical staging

^a^ Data mean ± standard deviation

^b^ Data are numbers of patients, with the percentage in parentheses

^c^ Data are presented as percentages with the number of patients in parentheses

### The intra-class correlation coefficient

ICCs between the two radiologists in the training set were shown in Table 2. The ICC for SAD was 0.833, which exceeded the coefficients for LAD (0.817), SD-MPR (0.803), and LD-MPR (0.793). The ICC for SAD measurement between the two radiologists in both the training and validation sets was excellent, with an ICC value of 0.847.

**Table 2:** ICC for SAD, LAD, SD-MPR, and LD-MPR of SCLNs on CT in training set

SCLNs	Axial				MPR			
	Radiologist 1	Radiologist 2	ICC	*p*-value	Radiologist 1	Radiologist 2	ICC	*p*-value
SD (mm)	6.7 ± 1.4	6.5 ± 1.5	0.833	< 0.001	7.5 ± 3.9	8.1 ± 3.5	0.803	< 0.001
LD (mm)	8.9 ± 2.5	9.4 ± 2.4	0.817	< 0.001	9.70 ± 4.3	10.2 ± 5.2	0.793	< 0.001

*SAD* short-axis diameter, *LAD* long-axis diameter, *SD-MPR* short diameter of multiplanar reconstruction, *LD-MPR* long diameter of multiplanar reconstruction, *SCLN* supraclavicular lymph node

### ROC analysis

**Fig. 3:**
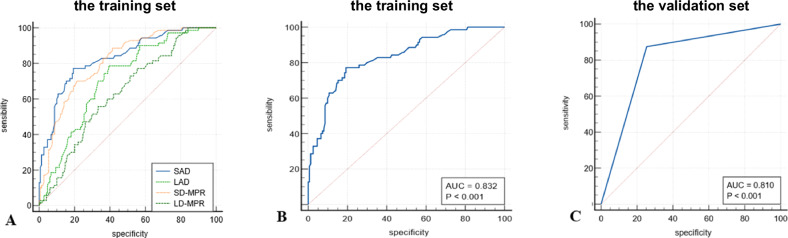
**A** ROC curve analysis for the training set showing the highest AUC value of 0.832 for SAD, exceeding those of LAD (0.711), SD-MPR (0.810), and LD-MPR (0.634). **B**, **C** ROC analysis of the SAD parameter for small SCLNs, the AUC of the training set is 0.832 and the AUC of the validation set is 0.810

In the training set, the efficacy of SAD, LAD, SD-MPR, and LD-MPR in detecting small SCLN metastases in ESCC was assessed. ROC curve analysis revealed that SAD yielded the highest AUC value of 0.832, surpassing the AUC values of LAD (0.711), SD-MPR (0.810), and LD-MPR (0.634) (Fig. 3A), with a threshold size criterion of > 6 mm, and the sensitivity, specificity, PPV, NPV, and accuracy were 77.1%, 80.7%, 65.0%, 88.3%, and 79.1%, respectively. Using the criterion of SAD > 6 mm, the AUC was 0.810, and the sensitivity, specificity, PPV, NPV, and accuracy were 87.5%, 74.5%, 61.8%, 92.7%, and 81.0% in the validation set, respectively (Fig. 3B, C).

### Lymph node analysis

In the training set, 70/220 (31.8%) were positive SCLNs; in the validation set, 24/75 (32%) were positive SCLNs.

The SAD of the positive SCLNs in the training set (mean = 7.7 mm, range 6.2–9.2 mm) and the validation set (mean = 7.9 mm, range 6.4–9.4 mm) showed statistically significant differences compared to that of the negative SCLNs (training set: mean = 5.8 mm, range 4.4–7.2 mm; validation set: mean = 5.4 mm, range 3.9–6.9 mm), as shown in Table 1.

In the training and validation sets, SCLNs with SAD < 5 mm accounted for 22% (49/220) and 27% (20/73), respectively, with metastasis rates of 6% (3/49) and 5% (1/20). Additionally, SCLNs with SAD of 5–10 mm accounted for 78% (171/220) and 73% (53/73), respectively, with metastasis rates of 39% (67/171) and 42% (23/55), as shown in Table 1.

## Discussion

This multicenter study demonstrated that the optimal threshold for predicting the metastatic status of SCLNs with SAD of < 10 mm in ESCC is 6 mm, with AUCs reaching 0.832 and 0.810 in the training and validation sets, respectively. The SAD on CT exhibited superior diagnostic performance in detecting small SCLN involvement, demonstrating higher AUC values compared to the LAD, SD-MPR, and LD-MPR. In the training cohort, we observed that CT measurement of SAD of SCLNs exhibited superior efficacy in diagnosing LN metastasis compared to LAD, SD-MPR and LD-MPR. These results align with the RECIST 1.1 guidelines, highlighting SAD as a more reliable metric for assessing LN involvement in clinical practice. We did not assess small LN volume measurements, as these typically require higher-precision imaging equipment and more complex image processing techniques, which could increase operational demands and costs, making them impractical for routine clinical use.

In our study, a minority of SCLNs with a SAD < 5 mm were found, 22% (49/220) and 27% (20/75) in training and validation sets, respectively. Among these SCLNs with a SAD < 5 mm, the proportion of metastatic SCLNs was 6% (3/49) and 5% (1/20) in training and validation sets, which was relatively low. This also indicated that SCLNs with a SAD < 5 mm are less likely to metastasize. In contrast, the majority of SCLNs, 78% (171/220) and 73% (55/75), had an SAD of 5–10 mm. Within this size category, the proportion of metastatic SCLNs was significantly higher, at 39% (67/171) and 42% (23/55).

The JEC-12 guidelines in 2024 proposed that a SAD of 6 mm on CT is recommended as the cutoff criterion, based on the analysis limited to regional LNs in stations No.101 cervical paraesophageal LNs, No.106 thoracic paratracheal LNs, No. 1 right paracardial LNs, No. 2 left paracardial LNs, and No. 3 lesser curvature LNs along the branches of the left gastric artery and lesser curvature LNs along the 2nd branches and distal part of the right gastric artery with SAD ≥ 5 mm of patients with cT2-T4 [5]; notably, SCLNs were not included in this study. In our study, we proposed a SAD of > 6 mm to evaluate SCLNs with SAD < 10 mm in ESCC, aligning with JEC-12 which analyzed LNs with SAD ≥ 5 mm in cT2-T4 patients (*N* = 224) [5]. Both studies obtained a consistent critical value of 6 mm, suggesting a possible universal relevance in the assessment criteria for SAD across different EC LN subregions. Compared to JEC-12, our study benefits from a larger sample size and multicenter study, enhancing statistical power and external validity. Additionally, it includes patients across all clinical T stages and those who were found to have small SCLNs for the first time, increasing generalizability and relevance. Distinctively, although both studies obtained the same cutoff values, their diagnostic performances differed. Our study demonstrates that when using SAD > 6 mm to diagnose metastasis SCLNs, the training and validation sets exhibited similar performance. Specifically, in the training set, the sensitivity, specificity, PPV, and NPV were 77.1%, 80.7%, 65.0%, and 88.3%, respectively, while in the validation set, these values were 87.5%, 74.5%, 61.8% and 92.7%. However, in JEC-12, the diagnostic performance significantly decreased. For instance, the results for No.101 cervical paraesophageal LNs and No.106 thoracic paratracheal LNs were 65.2%, 52.1%, 40.8%, and 74.7%, respectively, whereas the results for No.1 right paracardial LNs, No.2 left paracardial LNs, No.3 lesser curvature LNs along the branches of the left gastric artery and lesser curvature LNs along the 2nd branches and distal part of the right gastric artery, and No.7 LNs along the left gastric artery were 81.8%, 39.7%, 67.3%, and 59.0%, respectively. These discrepancies may be attributed to variations in LN anatomical locations, warranting further investigation for validation.

**Fig. 4:**
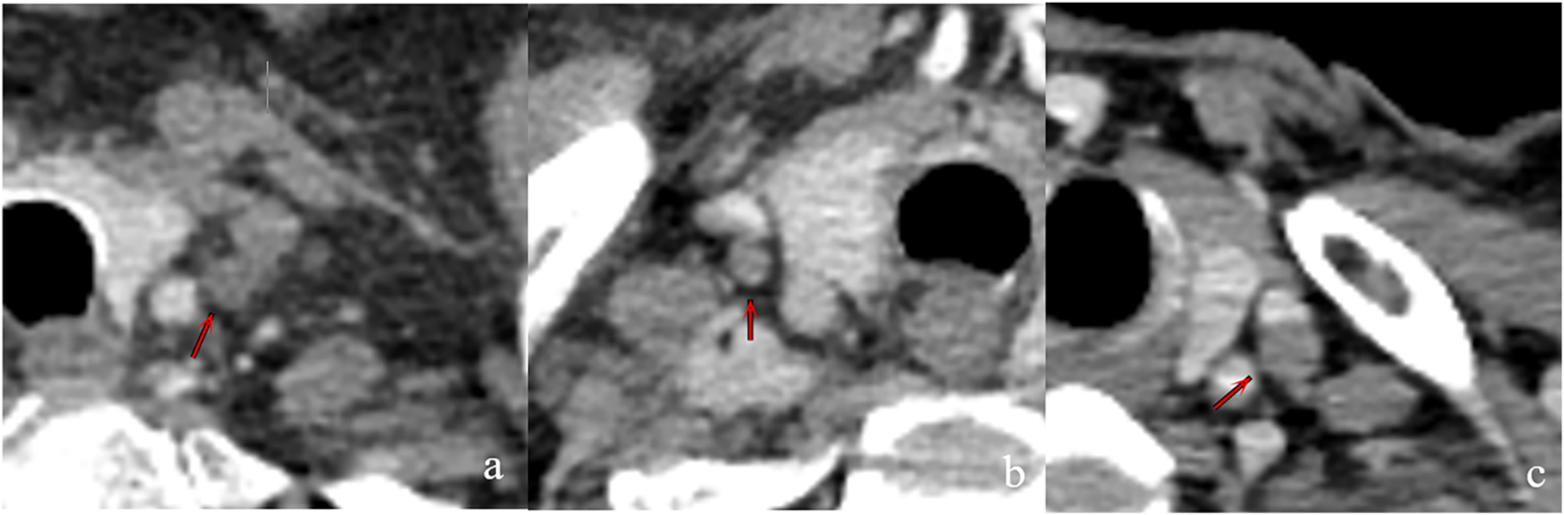
A 72-year-old male with ESCC. **a** Enhanced CT scan shows a small left SCLN with SAD of 5.8 mm (red arrow), exhibiting a heterogeneous configuration. Subsequent US-FNAB of the SCLN revealed negative pathological results. **b** A 73-year-old male with ESCC. Enhanced CT scan shows a small right SCLN with SAD of 4.7 mm, exhibiting a heterogeneous configuration. Subsequent US-FNAB of the SCLN revealed negative pathological results. **c** A 66-year-old male with ESCC. Enhanced CT scan shows a small left SCLN with SAD of 8.8 mm, exhibiting a heterogeneous configuration. Subsequent US-FNAB of the SCLN revealed positive pathological result

Elsholtz et al [28] addressed the lack of consensus in imaging evaluation of LN metastasis by introducing a size and structure-based LN scoring system. Generally, SAD ≥ 10 mm suggests metastasis. For certain anatomical areas, such as the face and parotid glands, the cutoff value for SAD is 5 mm, while it is 15 mm for the inguinal region. However, no specific cutoff value is mentioned for the supraclavicular area. In addition, the analysis of LN configuration is also crucial. CT has inherent limitations in soft tissue resolution. In our study, we analyzed 220 SCLNs from the training set and found that few exhibited a homogeneous internal structure on thin-section CT images; instead, most displayed pronounced heterogeneity (Fig. 4). Notably, only 16% (35 out of 220) of the SCLNs showed definite necrosis. Therefore, these findings suggest that for SCLNs with SAD < 10 mm, SAD measurements provide more valuable information than assessments of configuration. Therefore, this study primarily utilized SAD as the main evaluation parameter.

Some researchers also performed studies on LN metastases in ESCC, one meta-analysis of the two centers showed PET/CT demonstrates a relatively low sensitivity of 53–66% for diagnosing lymph node metastases in ESCC. This may be due to the difficulty in detecting a large number of small LN metastases, related to the impaired ability of small LNs to absorb F-18FDG [29, 30]. In a different study, Xie et al demonstrated that a two-dimensional radiomics-based model could non-invasively predict the metastatic status of individual LNs in ESCC [31], showing superior performance to traditional size-based measurement methods. However, the integration of imaging radiomics into clinical practice still faces significant challenges, including issues related to standardization and data complexity, which hinder its widespread adoption.

This study has some limitations. First, the histopathological information of SCLNs by US-FNAB was used as the gold standard, which may result in false negatives. Although the accuracy of US-FNAB for detecting SCLNs is 97% [12], it is still the recognized method in clinical practice. In this study, two excluded patients had insufficient cellularity to determine the nature of the lesions. Second, the detection of metastases in tiny SCLNs measuring ≤ 6 mm presents a challenge on CT scans, as they may not be easily distinguishable from nonmetastatic nodes, potentially leading to underestimation. Third, while SCLN metastasis is a significant prognostic indicator for patients with EC, its impact on survival outcomes cannot be overlooked. However, due to short follow-up time currently, we are unable to assess the influence of SCLN metastasis on patient survival. Future research will involve ongoing follow-up of these patients to evaluate the potential impact on survival outcomes. Finally, it is important to acknowledge the retrospective nature of this study, while it provided real world insights, further validation through extensive prospective studies is warranted to corroborate our findings.

In conclusion, our study demonstrated that the SAD measured on CT images was effective in evaluating small SCLN metastasis in patients with ESCC. With an optimal cutoff value of 6 mm, this approach enhances the accuracy of predicting SCLN metastasis, thereby improving diagnostic and therapeutic strategies for ESCC.

## Supplementary information

ELECTRONIC SUPPLEMENTARY MATERIAL

## Data Availability

The original data supporting the findings of this study are readily available upon request. They can be obtained from the corresponding author without any undue delay.

## Abbreviations

AUC: Area under the curve
EC: Esophageal carcinoma
ESCC: Esophageal squamous cell carcinoma
ICC: Intra-class correlation coefficient
LAD: Long-axis diameter
LD-MPR: Long diameter of multiplanar reconstruction
NPV: Negative predictive value
PPV: Positive predictive value
ROC: Receiver operating characteristics
SAD: Short-axis diameter
SCLN: Supraclavicular lymph node
SD-MPR: Short diameter of multiplanar reconstruction
US-FNAB: Ultrasound-guided fine-needle aspiration biopsy

## References

[1] Siegel RL, Miller KD, Wagle NS, Jemal A (2023) Cancer statistics, 2023. CA Cancer J Clin 73:17–48 36633525 10.3322/caac.21763

[2] Feng R-M, Zong Y-N, Cao S-M, Xu R-H (2019) Current cancer situation in China: good or bad news from the 2018 global cancer statistics? Cancer Commun 39:22–34 10.1186/s40880-019-0368-6 PMC6487510 31030667

[3] Boehme F, Racz K, Sebesta C Jr, Sebesta C (2023) Esophageal cancer. Wien Med Wochenschr 173:209–215 36318394 10.1007/s10354-022-00972-9

[4] Rice TW, Patil DT, Blackstone EH (2017) 8th edition AJCC/UICC staging of cancers of the esophagus and esophagogastric junction: application to clinical practice. Ann Cardiothorac Surg 6:119–130 28447000 10.21037/acs.2017.03.14 PMC5387145

[5] Doki Y, Tanaka K, Kawachi H et al (2024) Japanese classification of esophageal cancer, 12th edn: Part II. Esophagus 21:216–269 38512393 10.1007/s10388-024-01048-w PMC11199314

[6] Mine S, Tanaka K, Kawachi H et al (2024) Japanese classification of esophageal cancer, 12th edn: Part I. Esophagus 21:179–215 38568243 10.1007/s10388-024-01054-y PMC11199297

[7] Japan Esophageal S (2017) Japanese classification of esophageal cancer, 11th edn: Part II and III. Esophagus 14:37–65 28111536 10.1007/s10388-016-0556-2 PMC5222925

[8] Japan Esophageal S (2017) Japanese classification of esophageal cancer, 11th edn: Part I. Esophagus 14:1–36 28111535 10.1007/s10388-016-0551-7 PMC5222932

[9] Tachimori Y, Nagai Y, Kanamori N, Hokamura N, Igaki H (2011) Pattern of lymph node metastases of esophageal squamous cell carcinoma based on the anatomical lymphatic drainage system. Dis Esophagus 24:33–38 20626450 10.1111/j.1442-2050.2010.01086.x

[10] Tachimori Y, Ozawa S, Numasaki H et al (2014) Supraclavicular node metastasis from thoracic esophageal carcinoma: a surgical series from a Japanese multi-institutional nationwide registry of esophageal cancer. J Thorac Cardiovasc Surg 148:1224–1229 24613171 10.1016/j.jtcvs.2014.02.008

[11] Vanoverhagen H, Lameris JS, Zonderland HM, Tilanus HW, Vanpel R, Schutte HE (1991) Ultrasound and ultrasound-guided fine needle aspiration biopsy of supraclavicular lymph-nodes in patients with esophageal-carcinoma. Cancer 67:585–587 1985752 10.1002/1097-0142(19910201)67:3<585::aid-cncr2820670310>3.0.co;2-q

[12] Van Vliet EPM, Van Der Lugt A, Kuipers EJ et al (2007) Ultrasound, computed tomography, or the combination for the detection of supraclavicular lymph nodes in patients with esophageal or gastric cardia cancer: a comparative study. J Surg Oncol 96:200–206 17455243 10.1002/jso.20819

[13] Kutup A, Link BC, Schurr PG et al (2007) Quality control of endoscopic ultrasound in preoperative staging of esophageal cancer. Endoscopy 39:715–719 17661247 10.1055/s-2007-966655

[14] Sun Y, Sun Y, Li Z et al (2024) 18F-FAPI PET/CT performs better in evaluating mediastinal and hilar lymph nodes in patients with lung cancer: comparison with 18F-FDG PET/CT. Eur J Med Res 29:9–18 38173034 10.1186/s40001-023-01494-9 PMC10763273

[15] Eisenhauer EA, Therasse P, Bogaerts J et al (2009) New response evaluation criteria in solid tumours: revised RECIST guideline (version 1.1). Eur J Cancer 45:228–247 19097774 10.1016/j.ejca.2008.10.026

[16] Sohaib SA, Turner B, Hanson JA, Farquharson M, Oliver RTD, Reznek RH (2000) CT assessment of tumour response to treatment: comparison of linear, cross-sectional and volumetric measures of tumour size. Br J Radiol 73:1178–1184 11144795 10.1259/bjr.73.875.11144795

[17] Sgourakis G, Gockel I, Lyros O, Hansen T, Mildenberger P, Lang H (2011) Detection of lymph node metastases in esophageal cancer. Expert Rev Anticancer Ther 11:601–612 21504265 10.1586/era.10.150

[18] Alper F, Turkyilmaz A, Kurtcan S et al (2011) Effectiveness of the STIR turbo spin-echo sequence MR imaging in evaluation of lymphadenopathy in esophageal cancer. Eur J Radiol 80:625–628 20800403 10.1016/j.ejrad.2010.08.003

[19] Liu J, Wang Z, Shao H, Qu D, Liu J, Yao L (2018) Improving CT detection sensitivity for nodal metastases in oesophageal cancer with combination of smaller size and lymph node axial ratio. Eur Radiol 28:188–195 28677059 10.1007/s00330-017-4935-4

[20] Schröder W, Baldus SE, Mönig SP, Beckurts TKE, Dienes HP, Hölscher AH (2002) Lymph node staging of esophageal squamous cell carcinoma in patients with and without neoadjuvant radiochemotherapy: histomorphologic analysis. World J Surg 26:584–587 12098049 10.1007/s00268-001-0271-5

[21] van Vliet EPM, Heijenbrok-Kal MH, Hunink MGM, Kuipers EJ, Siersema PD (2008) Staging investigations for oesophageal cancer: a meta-analysis. Br J Cancer 98:547–557 18212745 10.1038/sj.bjc.6604200 PMC2243147

[22] Wang Y, Xiao P, Yang N et al (2021) Unresected small lymph node assessment predicts prognosis for patients with pT3N0M0 thoracic esophageal squamous cell carcinoma. World J Surg Oncol 19:303–316 34657600 10.1186/s12957-021-02412-1 PMC8522218

[23] Wang GM, Liu DF, Xu YP, Meng T, Zhu F (2016) PET/CT imaging in diagnosing lymph node metastasis of esophageal carcinoma and its comparison with pathological findings. Eur Rev Med Pharmacol Sci 20:1495–1500 27160120

[24] Choi J, Kim SG, Kim JS, Jung HC, Song IS (2010) Comparison of endoscopic ultrasonography (EUS), positron emission tomography (PET), and computed tomography (CT) in the preoperative locoregional staging of resectable esophageal cancer. Surg Endosc 24:1380–1386 20033712 10.1007/s00464-009-0783-x

[25] Li Z-X, Li X-D, Liu X-B et al (2020) Clinical evaluation of right recurrent laryngeal nerve nodes in thoracic esophageal squamous cell carcinoma. J Thorac Dis 12:3622–3630 32802441 10.21037/jtd-20-774 PMC7399419

[26] Vandenbrekel MWM, Castelijns JA, Stel HV et al (1991) Occult metastatic neck disease—detection with US and US-guided fine-needle aspiration cytology. Radiology 180:457–461 2068312 10.1148/radiology.180.2.2068312

[27] Koo TK, Li MY (2016) A guideline of selecting and reporting intraclass correlation coefficients for reliability research. J Chiropr Med 15:155–163 27330520 10.1016/j.jcm.2016.02.012 PMC4913118

[28] Elsholtz FHJ, Asbach P, Haas M et al (2021) Introducing the Node Reporting and Data System 1.0 (Node-RADS): a concept for standardized assessment of lymph nodes in cancer. Eur Radiol 31:6116–6124 33585994 10.1007/s00330-020-07572-4 PMC8270876

[29] Hu J, Zhu D, Yang Y (2018) Diagnostic value of ^18^F-fluorodeoxyglucose positron-emission tomography/computed tomography for preoperative lymph node metastasis of esophageal cancer: a meta-analysis. Medicine (Baltimore) 97:50–60 10.1097/MD.0000000000013722 PMC6319779 30558091

[30] Jiang C, Chen Y, Zhu Y, Xu Y (2018) Systematic review and meta-analysis of the accuracy of 18F-FDG PET/CT for detection of regional lymph node metastasis in esophageal squamous cell carcinoma. J Thorac Dis 10:6066–6067 30622778 10.21037/jtd.2018.10.57 PMC6297400

[31] Xie C, Hu Y, Han L, Fu J, Vardhanabhuti V, Yang H (2022) Prediction of individual lymph node metastatic status in esophageal squamous cell carcinoma using routine computed tomography imaging: comparison of size-based measurements and radiomics-based models. Ann Surg Oncol 29:8117–8126 36018524 10.1245/s10434-022-12207-7

