# Supplementary material for: Threshold size criterion to suspect malignant supraclavicular lymph node < 10 mm in esophageal cancer
Source: Insights Imaging. 2025 Mar 5;16:50. doi: 10.1186/s13244-025-01929-3 (PMC11883069; doi:10.1186/s13244-025-01929-3)
Supplement: Supplementary file 1 — ELECTRONIC SUPPLEMENTARY MATERIAL [file 13244_2025_1929_MOESM1_ESM.docx]

**Table S1.** CT scanning protocols and parameters for Center 1

| **Parameters** | **CT1** | **CT2** | **CT3** | **CT4** | **CT5** |
| --- | --- | --- | --- | --- | --- |
| Manufacture | GE Healthcare | Siemens Healthineers | Philips Healthcare | Philips Healthcare | United Imaging |
| Scanner type | Revolution CT | SOMATOM perspective CT | Brilliance iCT256 | IQon Spectral CT | uCT 760 |
| Detection rows | 128 | 64 | 128 | 64 | 128 |
| Tube voltage (kV) | 120 | 120 | 120 | 120 | 120 |
| Tube current (mA) | 300-400 | 300 | 300 | 300 | 300 |
| Rotation collimation(mm) | 256×0.625 | 64×0.6 | 128×1.0 | 64×0.625 | 64×0.625 |
| Matrix | 512×512 | 512×512 | 512×512 | 512×512 | 512×512 |
| FOV (cm) | 36-50 | 36-50 | 35-50 | 35-50 | 35-50 |
| Reconstruction section thickness (mm) | 0.625 | 1 | 1.25 | 1 | 1 |
| Reconstruction algorithm | Standard | Standard | Standard | Standard | Standard |
| Pitch | 0.992 | 0.85 | 0.993 | 0.925 | 0.9 |
| Numbers of scanned patients | 36 | 49 | 25 | 61 | 49 |

**Table S2.** CT scanning protocols and parameters for Center 2

| **Parameters** | **CT1** | **CT2** | **CT3** | **CT4** | **CT5** |
| --- | --- | --- | --- | --- | --- |
| Manufacture | GE Healthcare | Toshiba Medical Systems | Philips Healthcare | Siemens Healthineers | Philips Healthcare |
| Scanner type | Discovery CT750 HD | 64 slice multidetector row CT | Brilliance-64 | SOMATOM Definition Flash CT | Ingenuity CT Core 128 |
| Detection layers | 128 | 64 | 64 | 128 | 128 |
| Tube voltage(kV) | 120 | 120 | 120 | 120 | 120 |
| Tube current (mA) | 300 | 300 | 300 | 300 | 300 |
| Rotation collimation(mm) | 256×0.625 | 64×0.6 | 128×0.625 | 128×0.6 | 128×0.625 |
| Matrix | 512×512 | 512×512 | 512×512 | 512×512 | 512×512 |
| FOV (cm) | 36×50 | 35×50 | 35×50 | 36×50 | 35×50 |
| Reconstruction section thickness (mm) | 1.25 | 1 | 1.25 | 1.25 | 1.25 |
| Reconstruction algorithm | Standard | Standard | Standard | Standard | Standard |
| Pitch | 1.375 | 0.6 | 0.937 | 0.8 | 0.925 |
| Numbers of scanned patients | 11 | 9 | 7 | 10 | 6 |

**Table S3.** CT scanning protocols and parameters for Center 3

| **Parameters** | **CT1** | **CT2** | **CT3** |
| --- | --- | --- | --- |
| Manufacture | GE Healthcare | Toshiba Medical Systems | United Imaging |
| Scanner type | Revolution CT | Aquilion One TSX-301A | uCT 780 |
| Detection rows | 128 | 64 | 128 |
| Tube voltage(kV) | 120 | 120 | 120 |
| Tube current(mA) | 200-350 | 350 | 350 |
| Rotation collimation(mm) | 256×0.625 | 64×0.6 | 128×0.625 |
| Matrix | 512×512 | 512×512 | 512×512 |
| FOV (cm) | 36-50 | 35-50 | 35-50 |
| Reconstruction section thickness (mm) | 0.625 | 1 | 1 |
| Reconstruction algorithm | Standard | Standard | Standard |
| Pitch | 1.375 | 0.6 | 0.9 |
| Numbers of scanned patients | 12 | 13 | 7 |
